# Training Efficiency and Transfer Success in an Extended Real-Time Functional MRI Neurofeedback Training of the Somatomotor Cortex of Healthy Subjects

**DOI:** 10.3389/fnhum.2015.00547

**Published:** 2015-10-09

**Authors:** Tibor Auer, Renate Schweizer, Jens Frahm

**Affiliations:** ^1^MRC Cognition and Brain Sciences Unit, Cambridge, UK; ^2^Biomedizinische NMR Forschungs GmbH am Max-Planck-Institut für biophysikalische Chemie, Göttingen, Germany

**Keywords:** human, neurofeedback, real-time fMRI, motor cortex, somatosensory cortex

## Abstract

This study investigated the level of self-regulation of the somatomotor cortices (SMCs) attained by an extended functional magnetic resonance imaging (fMRI) neurofeedback training. Sixteen healthy subjects performed 12 real-time functional magnetic resonance imaging neurofeedback training sessions within 4 weeks, involving motor imagery of the dominant right as well as the non-dominant left hand. Target regions of interests in the SMC were individually localized prior to the training by overt finger movements. The feedback signal (FS) was defined as the difference between fMRI activation in the contra- and ipsilateral SMC and visually presented to the subjects. Training efficiency was determined by an off-line general linear model analysis determining the fMRI percent signal changes in the SMC target areas accomplished during the neurofeedback training. Transfer success was assessed by comparing the pre- and post-training transfer task, i.e., the neurofeedback paradigm without the presentation of the FS. Group results show a distinct increase in feedback performance (FP) in the transfer task for the trained group compared to a matched untrained control group, as well as an increase in the time course of the training, indicating an efficient training and a successful transfer. Individual analysis revealed that the training efficiency was not only highly correlated to the transfer success but also predictive. Trainings with at least 12 efficient training runs were associated with a successful transfer outcome. A group analysis of the hemispheric contributions to the FP showed that it is mainly driven by increased fMRI activation in the contralateral SMC, although some individuals relied on ipsilateral deactivation. Training and transfer results showed no difference between left- and right-hand imagery, with a slight indication of more ipsilateral deactivation in the early right-hand trainings.

## Introduction

Neurofeedback training provides subjects with information about the activation of a specific brain region in order to facilitate a learning process aiming at self-regulation of this targeted activation. The concept has successfully been applied in various clinical fields (Lubar and Shouse, 1976; Sterman and Egner, 2006; Arns et al., 2009; Tan et al., 2009; Kim and Birbaumer, 2014). Due to its clinical abundance and ease of use electroencephalography (EEG) is still the most widely used method to derive a feedback signal (FS) for training. The advent of functional magnetic resonance imaging (fMRI), which offers much better spatial accuracy across the entire brain, raised the question, if smaller anatomically or functionally circumscribed brain regions could be targeted with a suitable fMRI neurofeedback training (Yoo and Jolesz, 2002). Its feasibility was largely improved by the development of real-time (rt) fMRI (Cox et al., 1995; Lee et al., 1998; Voyvodic, 1999; Gembris et al., 2000), accomplishing image reconstruction and activation analysis within the acquisition time of a single-volumetric fMRI dataset. Despite the poor temporal resolution of fMRI and the 6- to 8-s latency of the underlying hemodynamic response, several studies demonstrated successful neurofeedback trainings in brain areas, such as the motor cortex (deCharms et al., 2004; Yoo et al., 2008; Berman et al., 2012; Chiew et al., 2012), the anterior cingulate cortex (Weiskopf et al., 2003; Hamilton et al., 2011), the amygdala (Posse et al., 2003; Zotev et al., 2011), the parahippocampal place area, the supplementary motor area (Weiskopf et al., 2004), the auditory cortex (Yoo et al., 2007), and the insular cortex (Caria et al., 2007, 2010; Johnston et al., 2010). Unfortunately, the proof-of-principle nature of most of these reports led to a considerable variation of paradigms and study designs which so far preclude a definite determination and generalization of critical elements for a successful real-time functional magnetic resonance imaging (rt-fMRI) neurofeedback training.

The present work includes a relatively large number of 16 subjects who participated in an extensive 4 weeks rt-fMRI neurofeedback training targeting the somatomotor cortex (SMC). Its primary goal was to go beyond the proof of feasibility, and to investigate the most relevant basic questions associated with the training itself as well as the associated brain circuits (manuscript submitted).

The SMC, and specifically the hand knob area, which is associated with hand and finger movements, was chosen for two reasons: first, because it has already been investigated with rt-fMRI neurofeedback (deCharms et al., 2004; Yoo et al., 2008; Berman et al., 2012; Chiew et al., 2012) and, second, because it has received considerable prospect for clinical applications, such as rehabilitation after motor cortex-related stroke and Parkinson (Birbaumer et al., 2008; Mihara et al., 2013; Subramanian et al., 2013; Yilmaz et al., 2015). Similar to other fMRI NFB studies using motor imagery (deCharms et al., 2004; Berman et al., 2012; Chiew et al., 2012), the targeted region of interest (ROI) was determined with a finger-tapping task to ensure that the cortical areas relevant to motor activity of the fingers, including the tightly linked and impartible somatosensation, were being trained.

Embedded in the general neurofeedback design were questions related to hand dominance and the hemispheric contributions to the FS. Each training session composed of two right-hand and two left-hand training runs, in which the right-handed subjects utilized imagery of the dominant right and the non-dominant left hand, respectively, to explore the possible influence of hand dominance on the neurofeedback training. Consideration of the SMC of both hemispheres seems essential since motor movement as well as somatosensory stimulation of the hand results in fMRI activation of the contralateral SMC and deactivation of the ipsilateral SMC (Allison et al., 2000; Nirkko et al., 2001; Hayashi et al., 2008). The FS presented in this study is defined as the difference between contra- and ispilateral SMC activation, similarly to Chiew et al. (2012). This reflects the specific activation pattern and provides insight into possible self-regulation differences of fMRI activation and deactivation. The additional task of attaining the bidirectional regulation (left SMC vs. right SMC for the right-hand training; right SMC vs. left SMC for the left-hand training) also excludes the influence of unspecific effects, such as attention and arousal (Scharnowski et al., 2015).

An early fMRI NFB study using motor imagery has shown that a learned increase in SMC activation can be achieved after three training sessions (deCharms et al., 2004) and more recent, a report about success within a single-training session (Yoo et al., 2008) could not be reproduced (Berman et al., 2012). Contrasting these studies, the present neurofeedback training consisted of 12 sessions spread over 4 weeks. It was not tailored for fast success, but to gain insight into the development of possible voluntary control during the neurofeedback training. Therefore, training efficiency was determined for each training run and analyzed for evaluating the entire training. In addition to training efficiency, the overall success of the neurofeedback training was determined in the transfer task, in which subjects performed the same task as in the neurofeedback training, but without receiving the neurofeedback signal. The successful transfer of the strategy used during the training into a similar situation but without feedback is an essential measure for clinical effectiveness and as a task and as a measure for success so far only used by Berman et al. (2012). The correlation between these two independent measures, the training efficiency and the transfer success then allows to estimate a potential transfer success based on the training efficiency.

## Materials and Methods

### Experimental setup

Seventeen healthy young adults (10 male, mean age 26 ± 3.3, range 20–31 years) underwent the neurofeedback training. Sixteen subjects were right handed, one subject showed ambidexterity at a laterality index of 20 [overall laterality index 79 ± 21, based on Edinburgh Inventory (Oldfield, 1971)]. The control group consisted of 16 demographically matched right-handed individuals (7 male, mean age 27 years ± 3.5, range 22–34 years, laterality index 87 ± 12) (see Table 2 for detailed subject information). All experimental procedures conformed fully the institutional guidelines and they were approved by the institutional Review Board. Written informed consent was obtained from all subjects before each MRI examination.

Subjects in the training group underwent 14 MRI examinations: 1 pre-training session, 12 training sessions, and 1 post-training session. The pre-training session consisted of a whole-brain structural *T*_1_-weighted MRI measurement, a fMRI measurement of bimanual finger movements (functional localizer) to delineate the target ROI for the training within the left and right SMC, and fMRI runs of left- and right-hand motor imagery without neurofeedback which were otherwise equivalent to the neurofeedback training runs. These “non-feedback” fMRI measurements assessed the subjects’ ability to control their SMC activities for each hand prior to the training and allowed for a quantification of the final transfer success by comparing respective fMRI signal changes before and after training (transfer task).

The 12 training sessions were spread over 4 weeks with three sessions per week, scheduled at the same time of the day on Monday, Wednesday, and Friday to ensure consistency. In each training session, two fMRI neurofeedback runs of right-hand training and two runs of left-hand training were conducted with randomized order. No training outside the scanner was performed.

The post-training session consisted of the same measurements as the pre-training session: whole-brain structural *T*_1_-weighted MRI, fMRI of overt finger movements, and one fMRI run of motor imagery without neurofeedback for each hand (transfer task). The subjects did not receive any additional instruction for the overt finger movement task (e.g., to pay extra attention or to employ their optimized strategy).

The control group only underwent the pre-training session and, after 4 weeks without any training, the post-training session.

### Magnetic resonance imaging

Magnetic resonance imaging was conducted at 3-T (Tim Trio, Siemens Healthcare, Erlangen, Germany) using a 12-channel head coil for signal reception. Structural whole-brain *T*_1_-weighted MRI involved a non-selective inversion-recovery 3D FLASH sequence (TR = 2530 ms, TE = 3.65 ms, flip angle 7°, TI = 1100 ms) at a nominal resolution of 1.3 mm × 1.0 mm × 1.3 mm. All fMRI measurements were based on a gradient-echo EPI sequence (TR = 2000 ms, TE = 36 ms, flip angle 70°) with 2 mm isotropic spatial resolution (22 slices, AC–PC orientation) yielding voxel sizes (8 mm^3^) far smaller than in previous fMRI-based neurofeedback studies (20–50 mm^3^) (Zotev et al., 2011; Weiskopf, 2012; Baecke et al., 2015; Blefari et al., 2015). Real-time data export (Weiskopf et al., 2005) allowed for the use of an in-house neurofeedback toolbox achieving online fMRI analysis (see below). In parallel, all images were stored in the standard data base and corrected for motion as supplied by the manufacturer (Siemens Healthcare, Erlangen, Germany). These images were used for off-line whole-volume analysis. For each subject, a single-whole-brain EPI measurement with the same orientation as the fMRI measurements was obtained (TR/TE = 7210/36 ms, flip angle 70°, 2 mm isotropic resolution, 80 slices) to optimize registration of the partial-brain fMRI measurements to the structural whole-brain scan. The individual field-of-view (FOV) and slice positions of the different MRI measurements of the pre-training session were stored and re-applied in all subsequent sessions (AutoAlign Scout, Siemens) to minimize the spatial difference between datasets.

### Functional localizer: overt finger movements

**Figure 1:**
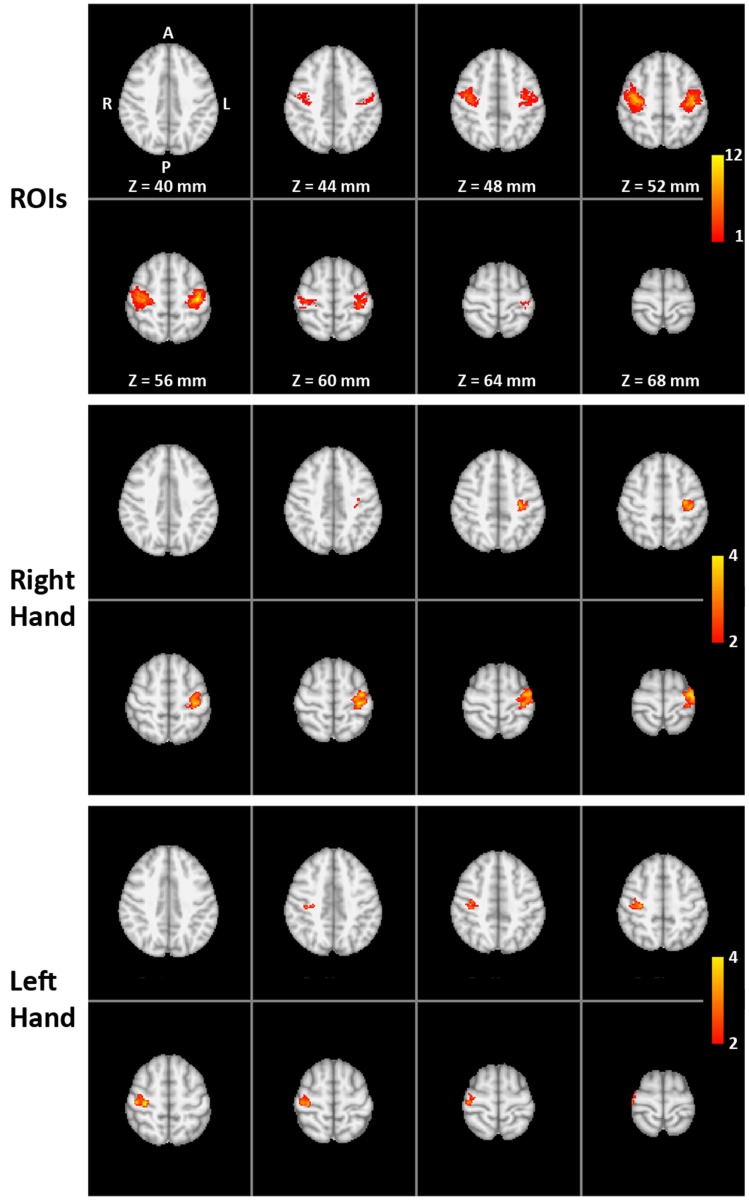
**Functional localization of the target ROIs**: (Top) overlap of individual target regions of the 16 trained subjects (MNI template) with colors indicating the number of subjects (1 to the maximum of 12) with suprathreshold activation during the pre-training overt finger movement task at a particular voxel (amount of overlap). (Middle and bottom) The two-way mixed ANOVA of the whole-brain volume for the right-hand (middle) and left-hand (bottom) transfer task without neurofeedback. Color indicates significantly higher pre- to post-training increase in activation for the training group compared to the control group (interaction TIME × GROUP).

Left and right SMC were identified individually based on fMRI of a bilateral sequential finger opposition task (Strother et al., 1995) comprising eight cycles of overt finger movements (12 s = 6 images) and motor rest (18 s = 9 images). Subjects were instructed to perform the finger task with both hands at a frequency of 1–2 Hz. Performance was monitored through a video surveillance system. The fMRI data were analyzed on a single-subject level using FEAT bundled in FSL 4.1.6 (FMRIB Center, Department of Clinical Neurology, University of Oxford, Oxford, UK). Preprocessing involved brain extraction, motion correction, and high-pass filtering, but no spatial filtering was applied to preserve the fine-scale spatial resolution. A general linear model (GLM) was then applied to the data with a double gamma hemodynamic response function. A temporal derivative was added to the design to increase robustness to a variable hemodynamic delay. Thresholding was accomplished by the two-threshold (TT) method, which does not require the assumption of a certain degree of spatial smoothness (Baudewig et al., 2003; Auer and Frahm, 2009). The upper threshold was set at *p* = 0.0001 and the lower threshold at *p* = 0.05. For each subject, significant activation clusters within left and right SMC (i.e., ROIs) were selected in native space (top of Figure 1 and Table 1).

**Table 1:** **Cluster extent (number of voxels) and coordinates (in mm, MNI space) for local maxima and centers of gravity within right and left somatomotor cortex (SMC)**.

Region	Number of voxels	Local maxima			Center of gravity		
	Mean ± SD	*X*	*Y*	*Z*	*X*	*Y*	*Z*
Left SMC	130 ± 34	−38 ± 5	−19 ± 6	54 ± 3	−37 ± 3	−19 ± 4	54 ± 2
Right SMC	144 ± 32	40 ± 4	−17 ± 6	52 ± 4	39 ± 3	−17 ± 4	52 ± 2

### fMRI neurofeedback training

Each fMRI training session consisted of four separate runs, two involving motor imagery of the left hand and two of the right hand. A training run started with a baseline period without a task (30 s = 15 images) and a control period (40 s = 20 images), followed by four cycles of training period (30 s = 15 images) and control period (40 s = 20 images) yielding a total duration of 5 min 50 s = 175 images. Short visual markers (500 ms) indicated the beginning and end of each training period. Subjects were instructed to find cognitive strategies that increase their brain activation in the SMC target regions. Examples of previously successful strategies were given for both training phases (e.g., imagining well-trained movements) and control phases (e.g., imagining landscapes or covert calculating). Subjects were also instructed to avoid deliberate changes in their general arousal state other than the given imagery task (deCharms et al., 2004) and to keep their breathing rate as constant as possible. It was strongly emphasized that any change had to be achieved without any overt movement. Absence of overt movement during motor imagery was verified by video surveillance (Lee et al., 2009).

During both training and control periods subjects received visual feedback via LCD goggles (VisuaStim XGA, Resonance Technology Inc., Northridge, CA, USA) with a latency of 8–10 s relative to the onset of neuronal activation, i.e., the hemodynamic latency, plus 2 s image acquisition, plus 1–2 s for real-time analysis. The feedback was presented by means of a horizontal blue rectangular bar (feedback meter) on a white screen. The bar was centered in the middle of the screen and its length changed toward the right or left side. For the right-hand training, the subjects’ task was to find a motor imagery strategy to increase the length of the bar to the right side, for the left-hand training to the left side of the screen. During the control periods, the feedback meter had to be kept as small as possible. During post-scanning interviews, possible improvements on the used strategy were discussed (e.g., imagining finger oppositions with random order and/or higher speed), but no training outside the scanner was asked for.

### fMRI neurofeedback signal processing

Real-time analysis and neurofeedback presentation were accomplished using an in-house neurofeedback toolbox implemented in MatLab (MathWorks, Natick, MA, USA). Each scan was automatically registered to the first scan of the overt finger movement task acquired in the pre-training session. Continuous motion correction was realized with real-time registration based on the SPM5 Realign function (Wellcome Trust Centre for Neuroimaging, University College London). For each of the two ROIs in left and right SMC, real-time percent signal change (rt%SC*)* was calculated for each time point with reference to the mean of the last 10 time points of the previous control period according to

(1) $$\text{rt}\%{\text{SC}}_{t}=\left(S_{\text{t}}/S_{\text{previous}\_\text{control}}-1\right)\times 100$$

where *S*_t_ and *S*_previous_control_ correspond to the signal intensity at time point *t* and during the previous control period, respectively.

To increase robustness and ensure insensitivity to the normalized signal fluctuations, a double logistic-like function with values ranging from 21 (for 2 rt%SC) to 0 (for −2 rt%SC) and a flat center between −0.25 and 0.25 rt%SC was applied. Similar to previous work (Lee et al., 2009), the FS given to the subjects was the difference between the real-time percent signal change from the left and right SMC:

(2) $${\text{FS}}_{\text{t}}=\text{rt}\%{\text{SC\_Left}}_{\text{t}}-\text{rt}\%\text{SC}\_{\text{Right}}_{\text{t}}$$

This resulted in a positive FS for successful right-hand training (right-sided elongation of the bar in the visual feedback) and a negative FS for successful left-hand training (elongation of the bar to the left side).

The training sessions were analyzed off-line using MatLab. GLM was performed on the time courses extracted from the individual ROIs used for training runs, and percent signal change for the contralateral (% signal change_contra_) and ipsilateral SMC (% signal change_ipsi_) were computed. Similar to Eq. 2, feedback performance (FP) of the subjects was defined as the signed difference between the percent signal changes of the SMC contra- and ipsilateral to the trained hand:

(3) $$\text{FP}=\%\text{ signal }{\text{change}}_{\text{contra}}-\%\text{ signal }{\text{change}}_{\text{ipsi}}$$

### Statistical analysis

#### Transfer Success

Transfer success was estimated using the fMRI data of the pre- and post-training fMRI measurements of motor imagery without neurofeedback (transfer task).

For the group analysis, FP (% signal change_contra_ − % signal change_ipsi_) values were entered in a three-way mixed ANOVA applying the within-subjects factors “HAND” (right- vs. left-hand training), “TIME” (pre- vs. post-training) and the between-subjects factor “GROUP” (training vs. control group).

For the analysis of the transfer success on the individual-subject level, the difference in pre- to post-training signal (% signal_post_ − % signal_pre_) in the left and right SMC ROI was determined in each subject for the right- and left-hand training separately. In the second step, the difference between this pre- to post-training change of the ipsi- and contralateral SMC (% signal change_contra_ − % signal change_ipsi_) was determined as an indicator for the transfer performance. These individual transfer success values were then tested for significant deviations against the pre- vs. post-training transfer measurement changes in the control group. Threshold values were calculated based on the one-sample *t*-test, taking the mean and SD of the transfer performance values from the pre- vs. post-training comparison of the control group as reference distribution and setting the value of the lower boundary of the confidence interval to *p* = 0.0032 based on a Bonferroni correction for multiple comparison for the 16 training subjects.

A voxel-wise fMRI analysis of the entire measured volume was also performed using FEAT. Preprocessing steps involved brain extraction, motion correction, high-pass filtering, and spatial filtering (FWHM = 5 mm) to allow for better registration (Maisog and Chmielowska, 1998) and to reduce within- and between-subject variability (Mikl et al., 2008). Because the fMRI datasets covered only part of the brain, a three-stage linear registration using FLIRT (Jenkinson and Smith, 2001) was performed to register the partial-volume images via the whole-brain images and the anatomical *T*_1_-weighted 3D images into standard MNI space. On the group level, a two-way mixed ANOVA with within-subjects factor “TIME” (pre- vs. post-training) and the between-subjects factor “GROUP” (training vs. control group) was performed. *Z* (Gaussianized *T*) statistic images were thresholded using clusters determined by *Z* > 2 and a cluster significance threshold of *p* = 0.05 corrected for multiple comparison.

#### Training Efficiency

Training efficiency was estimated comparing FP across training runs, including the pre-training ability transfer performance using a two-way within-subjects ANOVA with the factors “TIME” (pre-training transfer + 24 training runs) and “HAND” (left vs. right). *Post hoc* tests compared the FP (% signal change_contra_ − % signal change_ipsi_) of each of the 24 training runs to the performance (% signal change_contra_ − % signal change_ipsi_) during the pre-training transfer measurement, to investigate changes in the time course of the training compared to a baseline condition.

Changes in FP of two runs within a session (within session) and changes in mean FP of two consecutive sessions (between session) were also compared using two-way within-subjects ANOVA with the factors “INTERVAL” (within session vs. between session) and “HAND” (left vs. right) to investigate, which contributes more to the training effect.

To describe the efficiency of the single-training runs and the time course of the training in individual subjects, the FP of each of the 24 training of each subject runs was normalized (%SC training run − %SC pre-training run) and tested for a significant deviation against the pre- vs. post-training changes in the control group. Threshold values were calculated based on the one-sample *t*-test, taking the mean and SD of the FP values from the pre- vs. post-training comparison of the control group as reference distribution and setting the value of the lower boundary of the confidence interval to *p* = 0.0032 based on a Bonferroni correction for multiple comparison for the 16 training subjects. The sum of the training runs with significantly increased percent signal change was determined for each trained subject (number of efficient training runs).

#### Correlation of Training Efficiency and Transfer Success

To investigate the relationship between the training and the transfer, a number of Efficient Training Runs (see above) were correlated with the pre- to post-training change in FP.

#### Contributions of Contra- and Ipsilateral SMC to Feedback Signal

To investigate the separate contribution of the ipsi- and contralateral SMC (in% signal change) to the FP (combined measure from both hemispheres), the abovementioned ANOVAs were extended with an additional within-subject factor “HEMISPHERE” (contralateral vs. ipsilateral). Consequently, hemispheric contribution to the Training Success was analyzed using a four-way mixed ANOVA with within-subject factors “HAND” (left vs. right), “HEMISPHERE” (contralateral vs. ipsilateral), “TIME” (pre- vs. post-training transfer), and between-subject factor “GROUP” (training vs. control). Similarly, hemispheric contribution to the Training Efficiency was analyzed using a three-way within-subject ANOVA with factors “HAND” (left vs. right), “HEMISPHERE” (contralateral vs. ipsilateral), and “TIME” (pre-training transfer + 24 training runs).

#### Comparison of Right- and Left-Hand Trainings

The abovementioned ANOVAs also investigated the difference between the right- and the left-hand training (HAND effect). The length of the training was sufficient to reach a plateau showing small variability. Therefore, training courses were split in two halves, and the three-way within-subject ANOVA to test the hemispheric contribution to the Training Efficiency was extended with an additional within-subject factor “HALF” (first vs. second half).

Number of efficient contralateral SMC activations and ipsilateral SMC deactivations were also calculated similarly to the number of Efficient Training Runs, and they were entered in a two-way within-subject ANOVA (factors “HAND” and “HEMISPHERE”).

#### Overt Finger Movement Task

Pre- and post-training fMRI data obtained during the overt finger movement task were also analyzed in a way similar to the Training Success.

The average of the% signal change_contra_ and% signal change_ipsi_ values were entered in a two-way mixed ANOVA applying the within-subjects factor “TIME” (pre- vs. post-training) and the between-subjects factor “GROUP” (training vs. control group).

A voxel-wise fMRI analysis of the entire measured volume was also performed using FEAT and entered in a two-way mixed ANOVA again applying the within-subjects factor “TIME” (pre- vs. post-training) and the between-subjects factor “GROUP” (training vs. control group).

## Results

Of the 17 subjects that completed the training, 1 had to be excluded due to overt hand movement during the training. About 90% of the 192 training sessions were performed according to the planned schedule, on average only 1 session per subject had to be rescheduled.

### Training strategies

Post-training interviews revealed that subjects employed different motor imagery strategies, such as imagining playing an instrument, typing, squeezing the fist, or performing various handworks, such as knitting. Subjects reported a better performance when recalling the proprioceptive and motor aspect of the movement (i.e., kinesthetic motor imagery) rather than visualizing their moving fingers before their inner eye. They also stated that it was easier to alter the neurofeedback signal than to maintain it. For this latter purpose, subjects successfully modulated their preferred strategy by, for example, changing the speed or sequence of imagined movements. During the control periods, most participants tested several “passive” (e.g., relaxing) or “active” strategies (e.g., covertly singing or counting backward). “Active” control strategies turned out to be more successful.

### Transfer success

**Figure 2:**
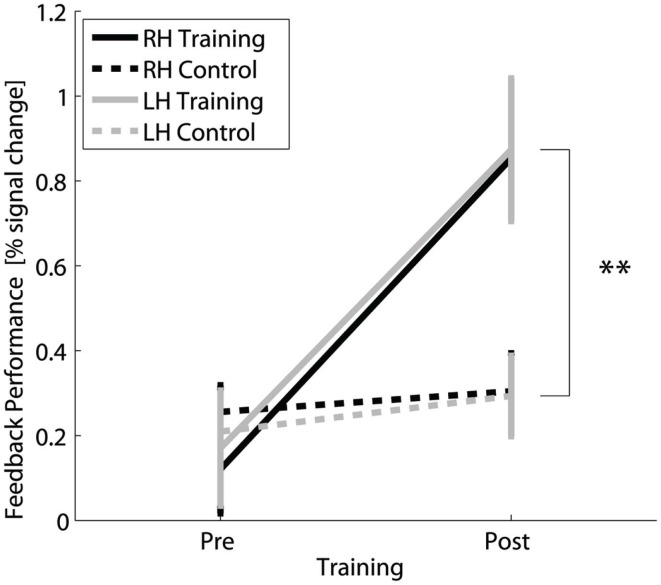
**Transfer success**: Feedback performance, i.e., difference in percent signal change in contra- vs. ipsilateral SMC, for fMRI of motor imagery without neurofeedback for the trained and control group for the right and left hand before and after 4 weeks of neurofeedback training. **Highly significant (*p* < 0.01) for both hands.

The successfulness of the training was based on the difference in fMRI activation during motor imagery without neurofeedback measured before and after the training compared to the difference signal from the control group measured before and after 4 weeks without training. The results show a clear distinction between the trained and control group for the interaction TIME × GROUP [*F*(1,30) = 23.5, *p* ≤ 0.001; *post hoc* contrast post trained vs. control *t*(30) = 3.4, *p* = 0.002 for right hand and *t*(30) = 3, *p* = 0.005 for left hand] (Figure 2) and main effect TIME [*F*(1,30) = 34.0, *p* ≤ 0.001]. The group of trained subjects presented a significant percent signal change increase in the SMC target regions (*post hoc* contrast trained subjects pre vs. post: *t*(15) = 5.7, *p* ≤ 0.001), whereas in the control group, no difference in percent signal change could be detected between the first and second measurement [*post hoc* contrast controls pre vs. post: *t*(15) = 1.4, n.s.]. Both the increase of percent signal change in trained subjects and the invariance in the controls are comparable for the right and left hand [Figure 2, main effect HAND: *F*(1,30) = 0.1, n.s.].

At the single-subject level, individual left- and right-hand fMRI changes (pre- to post-training) in the target regions of the trained subjects were compared to the respective data of the control group (Table 2). Eleven of the 16 subjects exhibited a significant percent signal increase in SMC for the left and right hand. Two subjects showed a significant increase only for the left or right hand. Three subjects showed no significant increase at all.

**Table 2:** **Trained (T) and control (C) subjects, difference in pre- to post-training percent signal change in somatomotor cortex (**Δ** SMC), and transfer success (TS)**.

Subject	Gender	Age	LI	Right-hand training			Left-hand training		
				Δ SMC L	Δ SMC R	TS	Δ SMC R	Δ SMC L	TS
C 01	Male	29	70	−0.01	−0.22	0.21	−0.31	−0.02	−0.29
C 02	Male	28	100	**0.37**	−**0.25**	**0.62**	**0.93**	0.84	0.09
C 03	Female	29	100	0.0	−0.10	0.10	0.14	0.07	0.07
C 04	Male	25	90	−0.23	−0.18	−0.05	−0.10	−**0.30**	0.20
C 05	Male	23	66	0.12	0.36	−0.24	0.03	0.30	−0.27
C 06	Male	23	85	**0.35**	0.20	0.15	**0.68**	0.53	0.14
C 07	Female	28	100	**0.55**	−0.01	**0.56**	−0.27	−**0.27**	−0.01
C 08	Female	24	100	−0.45	−0.02	−0.43	−0.37	−**0.36**	0.00
C 09	Female	31	90	−0.03	−0.15	0.13	**0.72**	0.14	**0.58**
C 10	Female	34	90	−0.25	−0.16	−0.09	0.11	−0.03	0.15
C 11	Male	25	80	−0.04	0.08	−0.13	0.05	0.32	−0.27
C 12	Female	22	80	−0.26	−0.15	−0.11	0.01	−0.12	0.13
C 13	Female	28	100	−0.19	−**0.28**	0.10	−0.21	−**0.46**	0.25
C 14	Female	24	85	0.25	0.16	0.10	0.27	−0.04	**0.31**
C 15	Male	30	70	0.15	−0.11	0.25	0.45	0.58	−0.13
C 16	Female	24	90	0.06	0.45	−0.40	0.46	0.08	**0.39**
Threshold*				**0.26**	−**0.21**	**0.31**	**0.51**	−**0.24**	**0.30**
T 01	Female	22	100	**0.49**	−**0.69**	**1.18**	0.25	−**0.38**	**0.64**
T 02	Female	26	85	**1.60**	0.24	**1.36**	**2.52**	0.42	**2.10**
T 03	Male	30	20	**0.60**	0.18	**0.43**	0.48	−0.02	**0.50**
T 04	Male	26	90	**1.53**	1.13	**0.40**	**2.03**	0.72	**1.31**
T 05	Female	30	60	**1.07**	−0.08	**1.15**	0.42	0.22	0.20
T 06	Male	21	90	**0.35**	0.41	−0.06	**1.52**	0.28	**1.24**
T 07	Male	26	100	**0.91**	0.37	**0.54**	**0.63**	0.00	**0.63**
T 08	Male	31	85	0.22	0.12	0.10	−0.22	−0.07	−0.15
T 09	Male	29	80	**2.02**	0.47	**1.55**	**1.35**	0.73	**0.62**
T 10	Female	25	90	−0.39	−**2.25**	**1.86**	0.01	−**1.19**	**1.20**
T 11	Female	28	55	0.22	−**0.52**	**0.74**	0.07	−0.24	**0.31**
T 12	Female	24	100	**0.50**	0.25	0.25	0.15	0.15	0.00
T 13	Male	27	65	**0.45**	−**0.23**	**0.67**	**0.81**	−0.18	**0.99**
T 14	Male	23	90	**0.56**	−**0.48**	**1.04**	0.33	−0.01	**0.35**
T 15	Female	20	70	−0.11	0.21	−0.31	0.26	0.26	0.00
T 16	Male	27	80	**1.38**	0.54	**0.84**	**2.22**	0.93	**1.30**

*LI, laterality index; L, left; R, right*.

*Bold numbers = significant pre- to post-training differences*.

*Underlined numbers = ipsilateral deactivation stronger than contralateral activation*.

*p = 0.05, Bonferroni corrected.

The two-way mixed ANOVA of the whole-brain fMRI data detected a significantly higher increase in activation for the training group than for the control group (interaction TIME × GROUP) only in the contralateral SMC for both right- and left-hand training (middle and lower parts of Figure 1). The locations of the clusters are comparable with the locations of the ROIs (upper part of Figure 1) indicating a high spatial specificity of the training effect.

### Training efficiency

**Figure 3:**
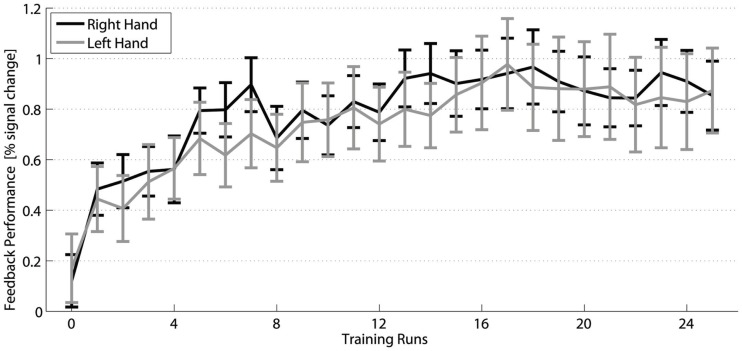
**Training efficiency**: Feedback performance (group mean **±** SEM) of the trained subjects across neurofeedback runs for the right (black) and left hand (gray).

The two-way within-subjects ANOVA (factors TIME and HAND) of the fMRI activation changes of the trained subjects across the 25 neurofeedback runs (1 pre-training run + 24 training runs) (Figure 3) showed a significant main effect TIME [*F*(24,360) = 9.3, *p* ≤ 0.001]. It corresponds to a significant increase of FP from the pre-training session to 15 out of the 24 training sessions (*post hoc* contrasts, *p* ≤ 0.05) and strongly indicates that the training of the SMC is effective in increasing the percent signal during the training.

**Figure 4:**
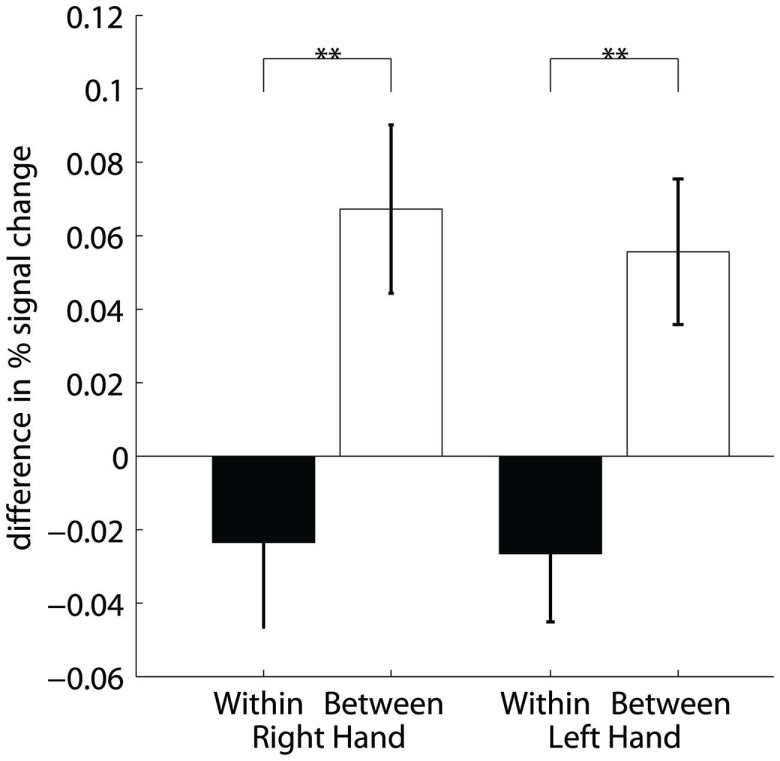
**Training efficiency**: Differences of percent signal change within sessions (two runs) and between session (2 days) for the right and left hand. **Highly significant (*p* < 0.01).

Figure 4 demonstrates that the increase in FP was significantly higher between training days (between sessions) than between runs (within sessions) [main effect INTERVAL *F*(1,191) = 17, *p* ≤ 0.001] regardless of the trained hand [non-significant main effect HAND *F*(1,191) = 0.1, *p* = 0.707]. Significant *post hoc* paired *t*-tests demonstrated an increase in FP between sessions compared to within sessions for both the right [*t*(191) = 3.2, *p* = 0.001] and the left [*t*(191) = 3.1, *p* = 0.002] hand. Further *post hoc* one-sample *t*-tests confirmed that within-session decreases were not significant [*t*(191) = −1, *p* = 0.309 and *t*(191) = −1.4, *p* = 0.154 for the right and the left hand, respectively]. On the contrary, between-session increases were significant [*t*(191) = 2.9, *p* = 0.004 and *t*(191) = 2.8, *p* = 0.006 for the right and the left hand, respectively].

**Figure 5:**
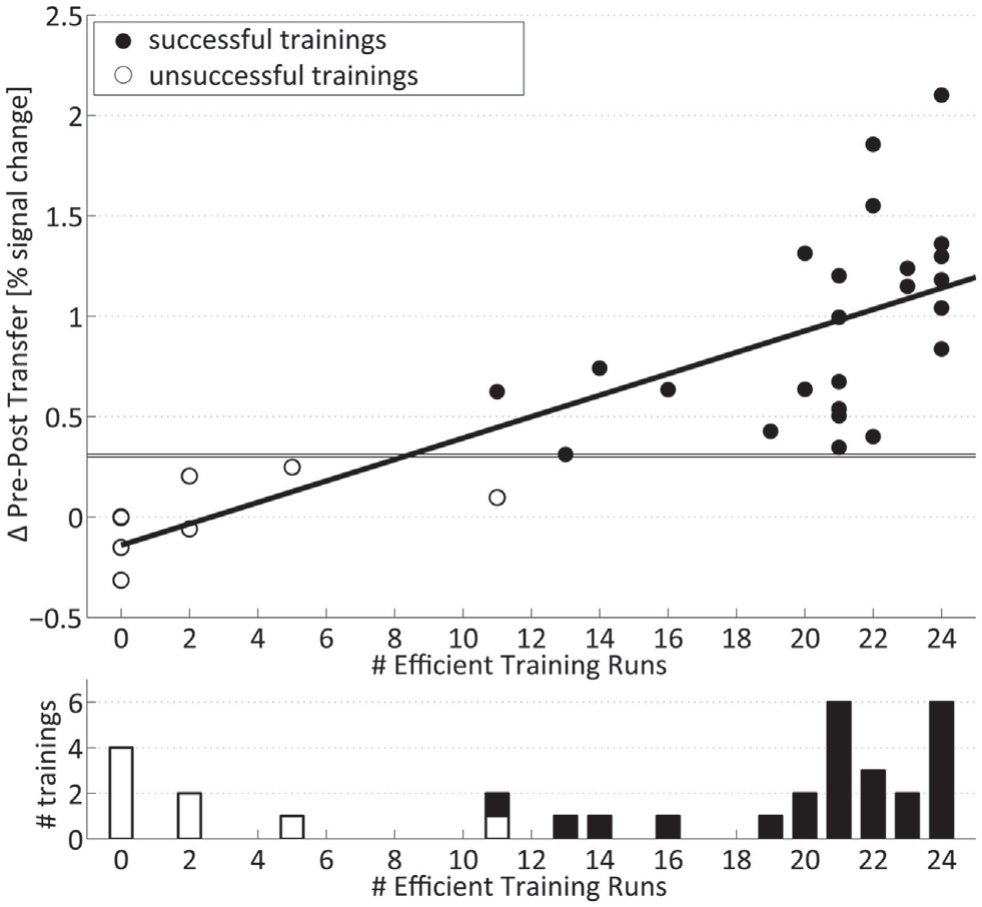
**Transfer success and training efficiency**: (Top) individual transfer success: values above horizontal lines indicate significance relative to controls for the right and left hand. (Bottom) number of subjects per summed number of training runs with significantly increased signal. Successfully trained subjects are marked with filled bars and circles.

Training efficiency on the single-subject level was described as the summed number of runs with increased activation for the left- and right-hand training. The distribution of the number of subjects per number of significantly increased training runs is shown in the lower part of Figure 5. The distribution is skewed to the two ends with a group of subjects with very few (<5 out of 24) significantly increased runs, a large group of subjects with a high number (more than 19) of increased runs, and a small in-between group with 11–16 significantly increased runs.

### Correlation of training efficiency and transfer success

The pre- to post-training fMRI signal increase during motor imagery without neurofeedback (transfer success) was highly significantly correlated (*r* = 0.78, *p* ≤ 0.001) (upper part of Figure 5) with the summed number of individual training runs with significantly increased signal change (training efficiency) and shows a negative intercept (Success = −0.14 + 0.05 × Efficiency). On the single-subject level, it can be seen that all trainings with a low number of individual runs with increased fMRI activation were associated with the lack of significant pre- to post-training changes in the motor imagery task (Table 2). On the other hand, all trainings with more than 19 significantly increased training runs showed a significant increase in percent signal change after 4 weeks. Figure 5 also demonstrates that trainings with intermediate numbers of significantly increased runs (i.e., 11–16) were also associated with a successful outcome. Taken together, subjects who are efficient in the training are also successful in the post-training transfer task, whereas subjects without efficient training are not.

### Contributions of contra- and ipsilateral SMC to feedback signal

**Figure 6:**
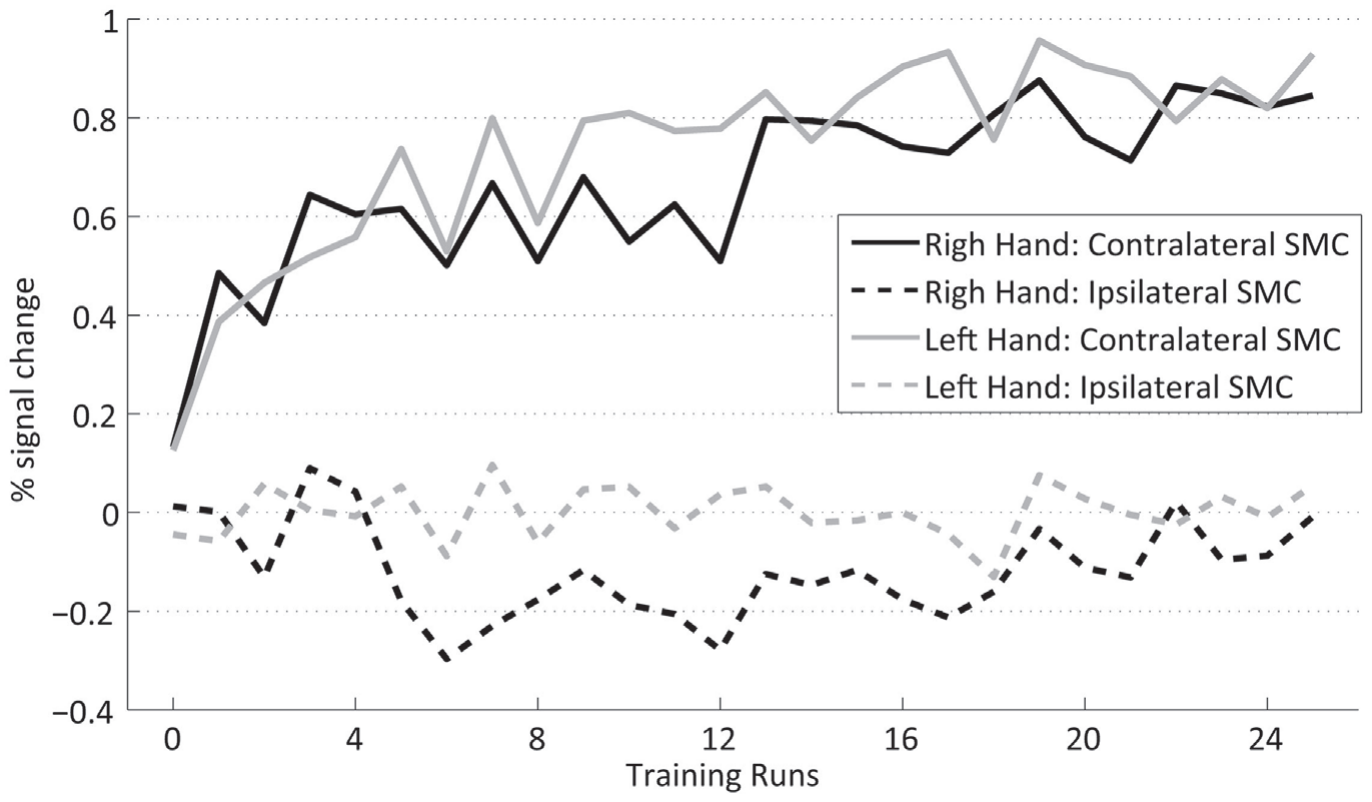
**Contralateral (dashed lines) and ispsilateral SMC (dotted lines) percent signal changes of the trained subjects across neurofeedback runs for the right (black) and left (gray) hand**.

Figure 6 presents the group average of the percent signal change for the ipsilateral and contralateral SMC for the right- and left-hand training. The contralateral hemisphere (solid lines) exhibits a larger percent signal increase (0.69 ± 0.147) than the ipsilateral hemisphere (dashed lines) (−0.061 ± 0.093) {main effect HEMISPHERE [*F*(1,15) = 50.4, *p* ≤ 0.001], whereas the significant interaction HEMISPHERE × TIME [*F*(24,360) = 9.3, *p* ≤ 0.001]} confirmed that this difference builds up during the time course of the training. This implies that the percent signal change increase during training is mainly due to increased fMRI activation in the contralateral SMC. This also holds true for the post-training transfer data [HEMISPHERE × GROUP × TIME *F*(1,30) = 23.4, *p* ≤ 0.001] (not shown) indicating that the GROUP × TIME interaction (i.e., transfer success) is larger in the contralateral SMC; therefore, the transfer success depended mainly on the increased fMRI signal in the contralateral SMC.

### Comparison of right- and left-hand trainings

The analysis of the transfer task revealed no difference between the signal changes achieved by training of the dominant right and the non-dominant left hand. Group analysis of the fMRI FP across the training shows a significant interaction HAND × TIME [*F*(1,15) = 6.2, *p* = 0.025, sixth training run, *post hoc* simple contrast] indicating a difference in the time course of the signal change between the hands across the training (Figure 3). The increased neurofeedback signal induced by the right-hand training can be explained by a complementary deactivation of the ipsilateral SMC, which was most pronounced during the first half of the right-hand training (Figure 6, black dashed line). During the left-hand training, no prominent deactivation of the ipsilateral SMC was observed, so that the differential neurofeedback signal is dominated by activation of the contralateral SMC (Figure 6, gray solid line). This difference does not reach significance in the overall analysis [HEMISPHERE × TIME × HAND *F*(24,360) = 0.6]. However, an explorative analysis just including the ipsilateral cortex, showed a significant main effect HAND [*F*(1,15) = 9.0, *p* = 0.009] as well as a significant interaction HAND × HALF × TIME [*F*(11,165) = 2.1, *p* = 0.019], which hints to a more prominent ipsilateral cortex deactivation for the dominant right hand during the first half of the training.

**Figure 7:**
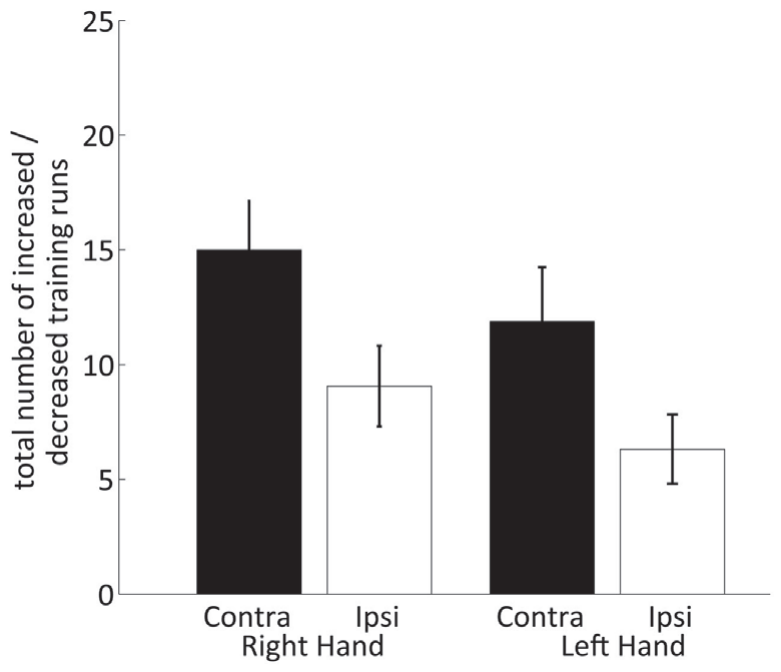
**Number of neurofeedback training runs with increased contralateral and decreased ipsilateral SMC percent signal changes for the right and left hand**.

The results of the two-way within-subject ANOVA of the hemispheric contribution to the efficiency (Figure 7) showed that runs with significantly decreased ipsilateral fMRI activations only occur with a significantly lower incidence than runs with significantly increased contralateral activation [HEMISPHERE *F*(1,15) = 17.3, *p* = 0.001]. Moreover, ipsilateral deactivation turned out to be somewhat more often during training of the right than left hand [non-significant trend *F*(1,15) = 3.3, *p* = 0.09].

Across the 16 trained subjects, 5 subjects showed a significant contribution of the ipsilateral cortex to the post-training neurofeedback signal (Table 2; two subjects for both hands, three subjects for the right hand only). Two of these subjects almost entirely relied on a deactivation in the ipsilateral SMC to achieve a positive neurofeedback signal.

### Overt finger movement task

**Figure 8:**
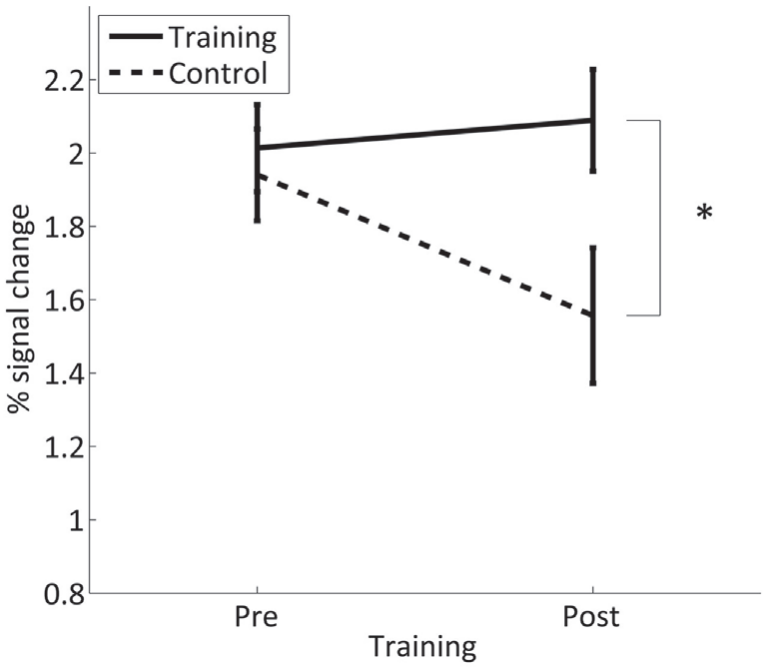
**Mean percent signal change in the contra- and ipsilateral SMC for overt finger movements during the pre- and post-training session in the trained and control group**. *Significant (*p* < 0.05).

The pre-training fMRI session involved overt movements of both hands to identify suitable ROIs in the SMC for the training. The task was repeated in the post-training session to assess if SMC activation in response to overt movement is altered by the neurofeedback training. In the control group, the analysis revealed a significant decrease in SMC activation [*t*(15) = 2.6, *p* = 0.021] from the first to the second measurement (after 4 weeks). By contrast, in the trained group, there is no such effect and SMC activation in the post-training session is significantly higher than in controls [*t*(15) = 2.5, *p* = 0.026] (Figure 8). The significant interaction GROUP × TIME [*F*(1,30) = 5.9, *p* = 0.021] confirms this difference between groups.

**Figure 9:**
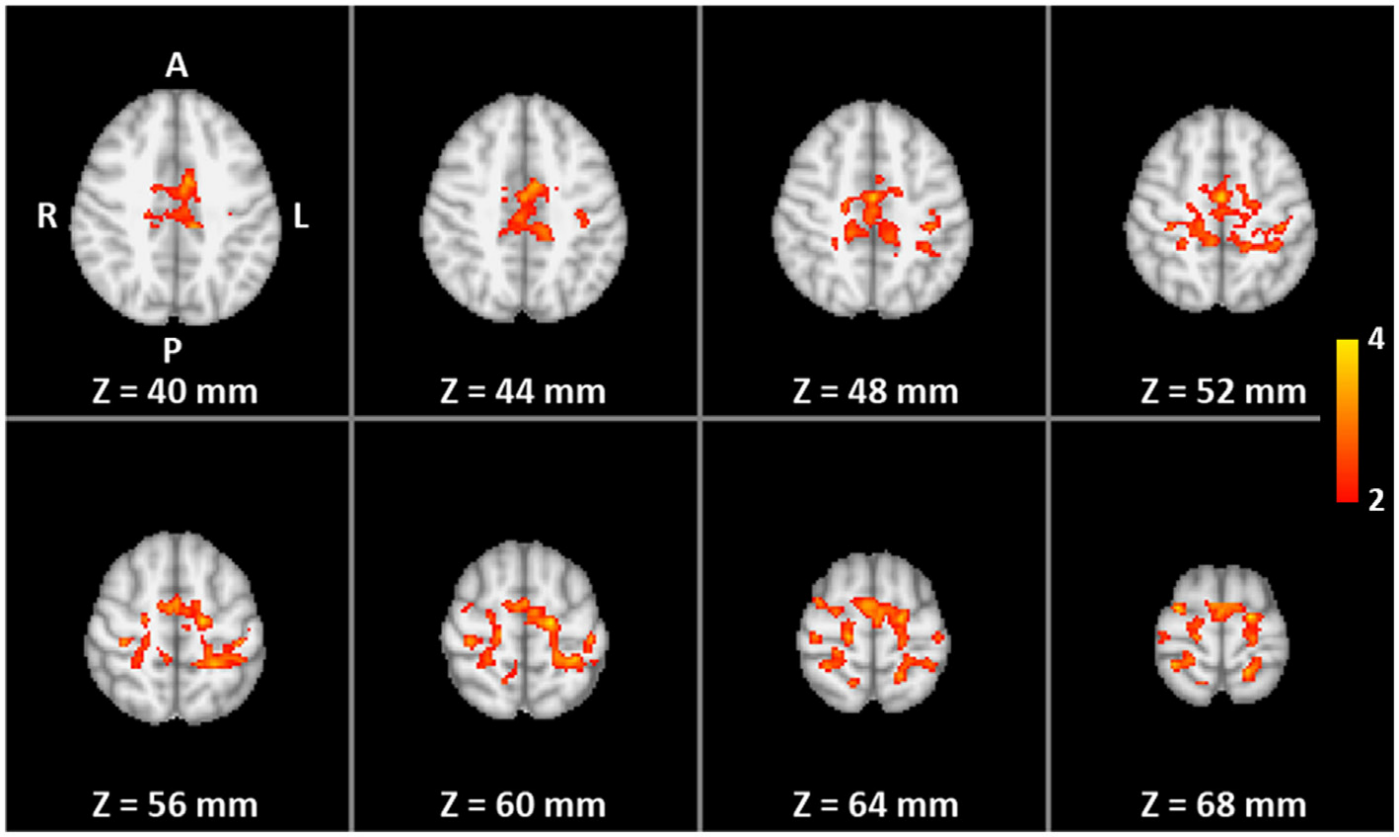
**The two-way mixed ANOVA of the whole volume for overt finger movements**. Color indicates significantly higher pre- to post-training increase in activation for the training group than for the control group (interaction TIME × GROUP).

A whole-brain analysis revealed that the training group shows additional significant differences in fMRI activation during the overt finger movement task before and after the training in the left primary somatomotor and premotor cortices as well as in the SMA, whereas the controls showed only a decrease in the abovementioned areas and in other regions, such as the superior parietal lobule. Both the increased activation in the training group and the decreased activation in the control group contribute to the significant interaction GROUP × TIME (Figure 9).

## Discussion

The present study investigated the degree of self-regulated control attainable for activation of the somatosensory-motor cortex by rt-fMRI neurofeedback training using motor imagery. More than two-thirds of the subjects in the training group did learn to efficiently increase the differential fMRI signal in more than three-quarter of the training runs. They were also successful in increasing activation in the post-training transfer task without receiving neurofeedback.

These results demonstrate, in line with many other studies (Caria et al., 2012; Weiskopf, 2012; Sulzer et al., 2013; Annette Beatrix, 2015), that fMRI neurofeedback is a suitable method to learn to deliberately modulate the fMRI response in a defined brain area. In addition, the remarkable spatial specificity of the accomplished signal increase after training, depicted in the sole activation of the SMC in the whole-brain analysis, argues against an unspecific learning effect. Self-regulation of brain activity was not only developed during the extended time course of the training but also reached the same magnitude during the post-training transfer task 2–3 days after the training, when the subjects performed the identical training paradigm without perceiving any neurofeedback. This condition is more demanding and requires a transfer of the obtained skill into a non-feedback situation, which can also be interpreted as a generalization of the training effect (Strehl et al., 2006). This observation is essential for the application of fMRI neurofeedback as a training strategy for long-lasting changes in cortical activation: either within clinical settings, such as in the neurorehabilitation of movement disabilities, or for the implementation of brain–computer interfaces, which rely on circumscribed and reproducible self-induced changes in activation (Birbaumer et al., 1999; Hatsopoulos and Donoghue, 2009).

In view of the aforementioned applications, the analysis of the neurofeedback training in a single subject comprises important information about the feasibility and predictability of the approach in the individual. One important measure is the failure rate. In the present study, one-fourth of all (7 out of 32 left and right hand) trainings were not successful. The reported failure rate for other SMC neurofeedback trainings greatly varies: 2 out of 11 subjects for 2 training sessions (Yoo et al., 2008), 7 out of 13 (Berman et al., 2012; Chiew et al., 2012) and 15 out of 15 subjects for 1 training session (Berman et al., 2012). Since the failure rate depends on many factors, including the brain area trained, the duration of the training, and the characteristics of the trained group of subjects, it remains difficult to compare results among studies with different paradigms. However, despite these limitations and under the consideration of the above mentioned factors, the failure rate might still be an appropriate measure to characterize the general feasibility of a trained task under the correspondent conditions and a starting point to explore the causes for non-performers (Weber et al., 2011).

The extended neurofeedback training of 12 sessions was set up to jointly address the dynamics of the learning process and the relationship between training efficiency and transfer success. The average learning curve of the group of trained subjects shows an increase of the neurofeedback signal during the initial phase and a leveling off in the last third of the training. But this group-averaged time course, comprising considerable variance, has to be interpreted with caution. Its layout reflects only a small minority of the individual learning curves, which show substantial variations. Individual learning curves range from early learners, which could either sustain efficiency to the end of the training or not, and if not, then even regain it at the end of the training, to very late learners. Because in general no consistent pattern of a learning time course could be detected across individuals, training runs with increased fMRI activation in the targeted SMC were solely added up across the course of the training to indicate training efficiency.

The linear correlation between the training efficiency and the transfer success clearly demonstrates that an efficient training generally leads to a successful transfer. The data also show a two-sided skewed distribution with non-learners, based on transfer task, realizing very few efficient training runs, at one end and the larger group of learners, achieving many efficient training runs, at the other end. The breaking point between learners and non-learners regarding the number of efficient training runs is around 12, all learners had 13 and more efficient training runs across the 24 training runs. This result is remarkably similar to the results of a recent EEG neurofeedback training study aiming to predict successful learning (Weber et al., 2011). By taking the increase of the amplitude of sensorimotor rhythms at mid-training (11 out of 25 trainings), the authors achieved a classification of performers and non-performers. These very similar observations suggest that the learning principles underlying EEG and fMRI neurofeedback are comparable and, despite the recording of very different brain signals, share the same neuronal basis. For fMRI-based neurofeedback trainings, the results would also imply a rule of thumb, that a successful transfer can be expected if at least half of the training runs reach a significantly increased activation. If that rule also holds for shorter trainings and other brain areas remains to be explored.

Another important result with respect to learning principles is the larger increase of the neurofeedback signal in the training runs across sessions on different days, rather than within sessions. This indicates a between days, off-training, consolidation effect, which should be considered in short-term neurofeedback trainings. Since the consolidating effect of sleep has been shown for motor skill (Walker et al., 2002; Sheth et al., 2008) and motor imagery learning (Debarnot et al., 2015), as well as in the cognitive and affective domain (Walker, 2009; Debarnot et al., 2013), it could be advantageous to distribute short neurofeedback trainings at least across different days.

The exploration of the influence of hand dominance on the neurofeedback training of the SMCs revealed no difference in the overall time course of the neurofeedback signal across training runs or in the transfer success between the left- and the right-hand training. Since the training or the transfer task did not incorporate any motor aspects, but mental imagery involving the left or right hand, this indicates that imagery, in contrast to executed motor behavior, was not influenced by hand dominance.

A slight indication for a difference between the left- and right-hand training was found in the hemispheric distribution of the contra- and ipsilateral SMC to the differential neurofeedback signal. Group analysis showed that the differential FS of the left- and right-hand training was mainly driven by fMRI activation in the contralateral SMC. A slight deviation from this pattern could be seen in the first half of the right-hand training, where fMRI deactivation in the ipsilateral cortex sustained the differential FS. Individual analysis revealed a relatively higher number of efficient right-hand trainings based on deactivation of the ipsilateral SMC, caused by a small number of individuals who relied on fMRI deactivation in the ipsilateral cortex to master the task contrasting the larger majority activating the contralateral SMC. The observation that self-regulation of the ipsilateral SMC is for some subjects feasible in the dominate right hand, could open up the possibility of clinical applications in specific cases, such as stroke in the somatomotor area, where the balance between the hemispheres can be disturbed (Ward and Cohen, 2004).

Finally, fMRI responses to the overt finger movement task before and after the neurofeedback training adds further information. In the group of trained subjects, fMRI activation in SMC elicited by the task in the post-training session reached the same level as in the pre-training session. This contrasts the results of the control group showing an actual decrease of the fMRI signal from the pre- to post-training finger movement task. A similar decrease has been described for overt wrist extension–flexion after a passive extension–flexion wrist training (Carel et al., 2000) and is interpreted as habituation to the repeated measurement of an identical motor task. The lacking of this habituation in the trained subjects may be a further generalization of the neurofeedback training into the overt movement condition, representing an activation of the trained SMCs. This generalization could be beneficial for neurofeedback applications, since it indicates that the self-regulation of a specific brain area learned from a training based on mental strategies can have generalized effects on the behavioral level, even if no direct self-regulation is applied.

## Conflict of Interest Statement

The authors declare that the research was conducted in the absence of any commercial or financial relationships that could be construed as a potential conflict of interest.

## References

[B1] Allison J. D. Meador K. J. Loring D. W. Figueroa R. E. Wright J. C. (2000). Functional MRI cerebral activation and deactivation during finger movement. Neurology 54, 135–142. 10.1212/WNL.54.1.135 10636139

[B2] Annette Beatrix B. (2015). Making sense of real-time functional magnetic resonance imaging (rtfMRI) and rtfMRI neurofeedback. Int. J. Neuropsychopharmacol. 18, yv020. 10.1093/ijnp/pyv020 25716778 PMC4438554

[B3] Arns M. De Ridder S. Strehl U. Breteler M. Coenen A. (2009). Efficacy of neurofeedback treatment in ADHD: the effects on inattention, impulsivity and hyperactivity: a meta-analysis. Clin. EEG Neurosci. 40, 180–189. 10.1177/155005940904000311 19715181

[B4] Auer T. Frahm J. (2009). Functional MRI using one- and two-threshold approaches in SPM5. Neuroimage 47, S102 10.1016/S1053-8119(09)70881-X

[B5] Baecke S. Lutzkendorf R. Mallow J. Luchtmann M. Tempelmann C. Stadler J. (2015). A proof-of-principle study of multi-site real-time functional imaging at 3T and 7T: implementation and validation. Sci. Rep. 5, 8413. 10.1038/srep08413 25672521 PMC4325335

[B6] Baudewig J. Dechent P. Merboldt K. D. Frahm J. (2003). Thresholding in correlation analyses of magnetic resonance functional neuroimaging. Magn. Reson. Imaging 21, 1121–1130. 10.1016/j.mri.2003.08.013 14725919

[B7] Berman B. D. Horovitz S. G. Venkataraman G. Hallett M. (2012). Self-modulation of primary motor cortex activity with motor and motor imagery tasks using real-time fMRI-based neurofeedback. Neuroimage 59, 917–925. 10.1016/j.neuroimage.2011.07.035 21803163 PMC3222744

[B8] Birbaumer N. Ghanayim N. Hinterberger T. Iversen I. Kotchoubey B. Kubler A. (1999). A spelling device for the paralysed. Nature 398, 297–298. 10.1038/18581 10192330

[B9] Birbaumer N. Murguialday A. R. Cohen L. (2008). Brain-computer interface in paralysis. Curr. Opin. Neurol. 21, 634–638. 10.1097/WCO.0b013e328315ee2d 18989104

[B10] Blefari M. L. Sulzer J. Hepp-Reymond M.-C. Kollias S. Gassert R. (2015). Improvement in precision grip force control with self-modulation of primary motor cortex during motor imagery. Front. Behav. Neurosci. 9:18. 10.3389/fnbeh.2015.00018 25762907 PMC4327737

[B11] Carel C. Loubinoux I. Boulanouar K. Manelfe C. Rascol O. Celsis P. (2000). Neural substrate for the effects of passive training on sensorimotor cortical representation[colon] a study with functional magnetic resonance imaging in healthy subjects. J. Cereb. Blood Flow Metab. 20, 478–484. 10.1097/00004647-200003000-00006 10724112

[B12] Caria A. Sitaram R. Birbaumer N. (2012). Real-time fMRI: a tool for local brain regulation. Neuroscientist. 18, 487–501. 10.1177/1073858411407205 21652587

[B13] Caria A. Sitaram R. Veit R. Begliomini C. Birbaumer N. (2010). Volitional control of anterior insula activity modulates the response to aversive stimuli. A real-time functional magnetic resonance imaging study. Biol. Psychiatry 68, 425–432. 10.1016/j.biopsych.2010.04.020 20570245

[B14] Caria A. Veit R. Sitaram R. Lotze M. Weiskopf N. Grodd W. (2007). Regulation of anterior insular cortex activity using real-time fMRI. Neuroimage 35, 1238–1246. 10.1016/j.neuroimage.2007.01.018 17336094

[B15] Chiew M. Laconte S. M. Graham S. J. (2012). Investigation of fMRI neurofeedback of differential primary motor cortex activity using kinesthetic motor imagery. Neuroimage 61, 21–31. 10.1016/j.neuroimage.2012.02.053 22401758

[B16] Cox R. W. Jesmanowicz A. Hyde J. S. (1995). Real-time functional magnetic resonance imaging. Magn. Reson. Med. 33, 230–236. 10.1002/mrm.1910330213 7707914

[B17] Debarnot U. Abichou K. Kalenzaga S. Sperduti M. Piolino P. (2015). Variable motor imagery training induces sleep memory consolidation and transfer improvements. Neurobiol. Learn. Mem. 119, 85–92. 10.1016/j.nlm.2014.12.010 25562401

[B18] Debarnot U. Piolino P. Baron J. C. Guillot A. (2013). Mental rotation: effects of gender, training and sleep consolidation. PLoS ONE 8:e60296. 10.1371/journal.pone.0060296 23544134 PMC3609807

[B19] deCharms R. C. Christoff K. Glover G. H. Pauly J. M. Whitfield S. Gabrieli J. D. E. (2004). Learned regulation of spatially localized brain activation using real-time fMRI. Neuroimage 21, 436–443. 10.1016/j.neuroimage.2003.08.041 14741680

[B20] Gembris D. Taylor J. G. Schor S. Frings W. Suter D. Posse S. (2000). Functional magnetic resonance imaging in real time (FIRE): sliding-window correlation analysis and reference-vector optimization. Magn. Reson. Med. 43, 259–268. 10.1002/(SICI)1522-2594(200002)43:2<259::AID-MRM13>3.0.CO;2-P 10680690

[B21] Hamilton J. P. Glover G. H. Hsu J. J. Johnson R. F. Gotlib I. H. (2011). Modulation of subgenual anterior cingulate cortex activity with real-time neurofeedback. Hum. Brain Mapp. 32, 22–31. 10.1002/hbm.20997 21157877 PMC3049174

[B22] Hatsopoulos N. G. Donoghue J. P. (2009). The science of neural interface systems. Annu. Rev. Neurosci. 32, 249–266. 10.1146/annurev.neuro.051508.135241 19400719 PMC2921719

[B23] Hayashi M. J. Saito D. N. Aramaki Y. Asai T. Fujibayashi Y. Sadato N. (2008). Hemispheric asymmetry of frequency-dependent suppression in the ipsilateral primary motor cortex during finger movement: a functional magnetic resonance imaging study. Cereb. Cortex 18, 2932–2940. 10.1093/cercor/bhn053 18413350 PMC2583153

[B24] Jenkinson M. Smith S. (2001). A global optimisation method for robust affine registration of brain images. Med. Image Anal. 5, 143–156. 10.1016/S1361-8415(01)00036-6 11516708

[B25] Johnston S. J. Boehm S. G. Healy D. Goebel R. Linden D. E. (2010). Neurofeedback: a promising tool for the self-regulation of emotion networks. Neuroimage 49, 1066–1072. 10.1016/j.neuroimage.2009.07.056 19646532

[B26] Kim S. Birbaumer N. (2014). Real-time functional MRI neurofeedback: a tool for psychiatry. Curr. Opin. Psychiatry 27, 332–336. 10.1097/YCO.0000000000000087 25023886

[B27] Lee C. C. Jack C. R. Rossman P. J. Riederer S. J. (1998). Real-time reconstruction and high-speed processing in functional MR imaging. AJNR Am. J. Neuroradiol. 19, 1297–1300. 9726472 PMC8332238

[B28] Lee J.-H. Ryu J. Jolesz F. A. Cho Z.-H. Yoo S.-S. (2009). Brain-machine interface via real-time fMRI: preliminary study on thought-controlled robotic arm. Neurosci. Lett. 450, 1–6. 10.1016/j.neulet.2008.11.024 19026717 PMC3209621

[B29] Lubar J. F. Shouse M. N. (1976). EEG and behavioral changes in a hyperkinetic child concurrent with training of the sensorimotor rhythm (SMR): a preliminary report. Biofeedback Self Regul. 1, 293–306. 10.1007/BF01001170 990355

[B30] Maisog J. M. Chmielowska J. (1998). An efficient method for correcting the edge artifact due to smoothing. Hum. Brain Mapp. 6, 128–136. 10.1002/(SICI)1097-0193(1998)6:3<128::AID-HBM2>3.0.CO;2-5 9673668 PMC6873362

[B31] Mihara M. Hattori N. Hatakenaka M. Yagura H. Kawano T. Hino T. (2013). Near-infrared spectroscopy-mediated neurofeedback enhances efficacy of motor imagery-based training in poststroke victims a pilot study. Stroke 44, 1091–1098. 10.1161/STROKEAHA.111.674507 23404723

[B32] Mikl M. Mareček R. Hluštík P. Pavlicová M. Drastich A. Chlebus P. (2008). Effects of spatial smoothing on fMRI group inferences. Magn. Reson. Imaging 26, 490–503. 10.1016/j.mri.2007.08.006 18060720

[B33] Nirkko A. C. Ozdoba C. Redmond S. M. Burki M. Schroth G. Hess C. W. (2001). Different ipsilateral representations for distal and proximal movements in the sensorimotor cortex: activation and deactivation patterns. Neuroimage 13, 825–835. 10.1006/nimg.2000.0739 11304079

[B34] Oldfield R. C. (1971). The assessment and analysis of handedness: the Edinburgh inventory. Neuropsychologia 9, 97–113. 10.1016/0028-3932(71)90067-4 5146491

[B35] Posse S. Fitzgerald D. Gao K. Habel U. Rosenberg D. Moore G. J. (2003). Real-time fMRI of temporolimbic regions detects amygdala activation during single-trial self-induced sadness. Neuroimage 18, 760–768. 10.1016/S1053-8119(03)00004-1 12667853

[B36] Scharnowski F. Veit R. Zopf R. Studer P. Bock S. Diedrichsen J. (2015). Manipulating motor performance and memory through real-time fMRI neurofeedback. Biol. Psychol. 108, 85–97. 10.1016/j.biopsycho.2015.03.009 25796342 PMC4433098

[B37] Sheth B. R. Janvelyan D. Khan M. (2008). Practice makes imperfect: restorative effects of sleep on motor learning. PLoS ONE 3:e3190. 10.1371/journal.pone.0003190 18787652 PMC2527676

[B38] Sterman M. B. Egner T. (2006). Foundation and practice of neurofeedback for the treatment of epilepsy. Appl. Psychophysiol. Biofeedback 31, 21–35. 10.1007/s10484-006-9002-x 16614940

[B39] Strehl U. Leins U. Goth G. Klinger C. Hinterberger T. Birbaumer N. (2006). Self-regulation of slow cortical potentials: a new treatment for children with attention-deficit/hyperactivity disorder. Pediatrics 118, e1530–e1540. 10.1542/peds.2005-2478 17060480

[B40] Strother S. C. Anderson J. R. Schaper K. A. Sidtis J. J. Liow J. S. Woods R. P. (1995). Principal component analysis and the scaled subprofile model compared to intersubject averaging and statistical parametric mapping: I. “Functional connectivity” of the human motor system studied with [15O]water PET. J. Cereb. Blood Flow Metab. 15, 738–753. 10.1038/jcbfm.1995.94 7673369

[B41] Subramanian L. Turner D. Morris H. Busse M. Linden D. (2013). Real-time fMRI neurofeedback for the treatment of Parkinson’s disease (PD). Mov. Disord. 28, S168–S169. 10.1002/mds.25605

[B42] Sulzer J. Haller S. Scharnowski F. Weiskopf N. Birbaumer N. Blefari M. L. (2013). Real-time fMRI neurofeedback: progress and challenges. Neuroimage 76, 386–399. 10.1016/j.neuroimage.2013.03.033 23541800 PMC4878436

[B43] Tan G. Thornby J. Hammond D. C. Strehl U. Canady B. Arnemann K. (2009). Meta-analysis of EEG biofeedback in treating epilepsy. Clin. EEG Neurosci. 40, 173–179. 10.1177/155005940904000310 19715180

[B44] Voyvodic J. T. (1999). Real-time fMRI paradigm control, physiology, and behavior combined with near real-time statistical analysis. Neuroimage 10, 91–106. 10.1006/nimg.1999.0457 10417244

[B45] Walker M. P. (2009). The role of sleep in cognition and emotion. Ann. N. Y. Acad. Sci. 1156, 168–197. 10.1111/j.1749-6632.2009.04416.x 19338508

[B46] Walker M. P. Brakefield T. Morgan A. Hobson J. A. Stickgold R. (2002). Practice with sleep makes perfect: sleep-dependent motor skill learning. Neuron 35, 205–211. 10.1016/S0896-6273(02)00746-8 12123620

[B47] Ward N. S. Cohen L. G. (2004). Mechanisms underlying recovery of motor function after stroke. Arch. Neurol. 61, 1844–1848. 10.1001/archneur.61.12.1844 15596603 PMC3713312

[B48] Weber E. Koberl A. Frank S. Doppelmayr M. (2011). Predicting successful learning of SMR neurofeedback in healthy participants: methodological considerations. Appl. Psychophysiol. Biofeedback 36, 37–45. 10.1007/s10484-010-9142-x 21053066

[B49] Weiskopf N. (2012). Real-time fMRI and its application to neurofeedback. Neuroimage 62, 682–692. 10.1016/j.neuroimage.2011.10.009 22019880

[B50] Weiskopf N. Klose U. Birbaumer N. Mathiak K. (2005). Single-shot compensation of image distortions and BOLD contrast optimization using multi-echo EPI for real-time fMRI. Neuroimage 24, 1068–1079. 10.1016/j.neuroimage.2004.10.012 15670684

[B51] Weiskopf N. Mathiak K. Bock S. W. Scharnowski F. Veit R. Grodd W. (2004). Principles of a brain-computer interface (BCI) based on real-time functional magnetic resonance imaging (fMRI). IEEE Trans. Biomed. Eng. 51, 966–970. 10.1109/TBME.2004.827063 15188865

[B52] Weiskopf N. Veit R. Erb M. Mathiak K. Grodd W. Goebel R. (2003). Physiological self-regulation of regional brain activity using real-time functional magnetic resonance imaging (fMRI): methodology and exemplary data. Neuroimage 19, 577–586. 10.1016/S1053-8119(03)00145-9 12880789

[B53] Yilmaz O. Birbaumer N. Ramos-Murguialday A. (2015). Movement related slow cortical potentials in severely paralyzed chronic stroke patients. Front. Hum. Neurosci. 8:1033. 10.3389/fnhum.2014.01033 25642177 PMC4295525

[B54] Yoo S.-S. Jolesz F. A. (2002). Functional MRI for neurofeedback: feasibility study on a hand motor task. Neuroreport 13, 1377–1381. 10.1097/00001756-200208070-00005 12167756

[B55] Yoo S. S. Lee J. H. O’Leary H. Lee V. Choo S. E. Jolesz F. A. (2007). Functional magnetic resonance imaging-mediated learning of increased activity in auditory areas. Neuroreport 18, 1915–1920. 10.1097/WNR.0b013e3282f202ac 18007186

[B56] Yoo S.-S. Lee J.-H. O’Leary H. Panych L. P. Jolesz F. A. (2008). Neurofeedback fMRI-mediated learning and consolidation of regional brain activation during motor imagery. Int. J. Imaging Syst. Technol. 18, 69–78. 10.1002/ima.20139 19526048 PMC2630170

[B57] Zotev V. Krueger F. Phillips R. Alvarez R. P. Simmons W. K. Bellgowan P. (2011). Self-regulation of amygdala activation using real-time FMRI neurofeedback. PLoS ONE 6:e24522. 10.1371/journal.pone.0024522 21931738 PMC3169601

